# Health conditions contribution to disability burden in Spain and the role of ethnicity and migrant status: A nation-wide study

**DOI:** 10.1371/journal.pone.0306526

**Published:** 2024-07-12

**Authors:** Javier Casillas-Clot, Pamela Pereyra-Zamora, Andreu Nolasco

**Affiliations:** Department of Community Nursing, Preventive Medicine, Public Health, and History of Science, Research Unit for the Analysis of Mortality and Health Statistics, University of Alicante, San Vicente del Raspeig, Spain; University of Coimbra: Universidade de Coimbra, PORTUGAL

## Abstract

**Background:**

Disability is frequently associated with contextual or lifestyle factors. Some health conditions may affect the prevalence of disability differently, especially for some minority groups. This study aims to assess the impact and contribution of different health conditions to disability burden in Spain in Roma and immigrant populations, compared to the general population.

**Methods:**

This is a cross-sectional study. We have used data from the Spanish National Survey of 2017 and the National Health Survey of the Roma Population 2014. We have calculated frequencies of demographic variables and prevalence of health conditions grouped by body function. We also have fitted binomial additive hazard models, using the attribution method, to assess disabling impact and contribution of health conditions to disability burden. The software R was used for the computations.

**Results:**

Roma and immigrant populations had worse socioeconomic status than the general population, although the gap was more heavily marked among Roma. Roma population showed a higher prevalence in all health conditions, with a disability prevalence of 57.90%, contrary to the immigrant population, that showed a lower prevalence in all health conditions, including disability (30.79%), than the general population (40.00%). However, all health conditions were more disabling in the immigrant population. Neurological and cardiovascular diseases, and accidents among Roma, were the most disabling conditions. Nevertheless, musculoskeletal, chronic pain, and sensory diseases among Roma, had a greater contribution to disability burden, mainly due to a combination of a great prevalence and a great impact in functions of those health conditions.

**Conclusion:**

Both ethnicity and migrant status have shown differences in the burden of disability. While in the general population, musculoskeletal problems have the greatest contribution to the disability burden, in immigrants it was chronic pain and in the Roma population it was sensory problems. Disparities by sex were also found, with the contribution of musculoskeletal diseases being more important in females.

## Introduction

The International Classification of Functioning (ICF) considers disability as a combination of impairments, activity limitations and participation restrictions [1]. It is estimated that 3,434,862 people with an administratively recognized disability lived in Spain in 2020 [2]. However, the figures may vary depending on the disability indicator used. Due to the lack of a gold standard to measure disability, different instruments, such as the Global Activity Limitation Index (GALI) [3], have been proposed [4].

Chronic conditions, such as musculoskeletal, cardiovascular and respiratory disease have been found to be frequent contributors to the burden of disability [5–7]. The main contributors are also usually the more prevalent conditions, while other health problems like strokes or neurological diseases could have a greater disabling impact [8,9]. However, differences have been found between countries and social groups, including differences by gender, age and educational level [5,7,10]. Minority groups, such as immigrants and Roma people, are expected to have different contributors in the burden of disability, since they have shown to have different disability determinants [11].

While some health conditions could be the causes of an impairment, it is the interaction with contextual factors (such as environment or personal lifestyles) which will lead to a disability status [1]. Indeed, factors such as gender, age, socioeconomic status or access to health system may determine the disabling impact of a health condition [12]. Structural racism and xenophobia are interconnected and lead to health inequities through different mechanisms [13]. Although in several cases immigrant populations have shown a better health status than host populations, the health status of this populations tends to deteriorate over time and in the following generations [11,14]. In fact, it is known that immigrants are more likely to accept jobs with a greater injury and health risk [15] and that they have less access to the health system [16]. In the Roma population’s case, the largest ethnic group in Spain, they are one of the most socially excluded groups in the country [17,18]. As a consequence of this situation they have a worse status in most health areas than the general population, including disability [11,19].

There is a long tradition in the study of the intersectionality of ethnicity, migration, and disability in the United States. However, very few studies have been conducted in Europe about this topic, although ethnicity and migration are greatly different in both continents [20]. In Spain most migrants come from north Africa or South America, due to geographical and cultural reasons, which makes Spanish migration unique [21]. Considering immigration represents almost the 15% of the Spanish population, it would be expected to have a great influence on the global burden of disability. On the other hand, the largest ethnic minority in Spain, the Roma people, has been inhabiting Spain from 500 years ago [22]. It is estimated that between 700,000 and 970,000 Roma (between 1.2% and 2.1% of Spanish population) with Spanish nationality could be living in Spain (which makes the Roma population in Spain one of the largest in the world) [23,24]. In view of all of this, it could be hypothesized that Spain’s particular ethnic diversity could lead to differences in disability and disease patterns with respect to other countries.

The aim of this study is to estimate the contribution of the health conditions to disability burden, defined as the prevalence of disability (using the GALI as a measure of disability), in immigrant and Roma populations residing in Spain and in the native population (Spanish-born population).

## Materials and methods

### Study design and sources

This is a cross-sectional study. Data from the Spanish population aged 50 years and above were used from the Spanish National Health Survey 2017 (ENSE) and the National Health Survey of the Roma Population 2014 (ENSPG), both provided by the Spanish Ministry of Health.

The ENSE is a nationally representative health survey. The total sample of the ENSE-2017 included 23,089 participants, selected using a stratified three-phase random sampling. Data collection was carried out through face-to-face interviews in the households of the respondents, using standardized questionnaires covering 4 areas: sociodemographic, health status, use of health services and health determinants. The data were segregated into population born in Spain (native or general population) and population born outside Spain (immigrant population). As inclusion criteria, participants had to be older than 50, so a total sample of 10,668 participants (9,879 of the native population and 790 of the immigrant population) was finally included in this study.

ENSPG is also a nationally representative heath survey with the difference that the sample universe only includes the Roma population. The total sample of the ENSPG-2014 consisted of 1,167 participants, selected using a stratified three-phase random sampling. The three phases of sampling were first at the census tract level, then at the household level, and finally at the individual level. A random sampling at each of these levels serves to ensure the correct representativeness of the population in the entire territory. Data collection was carried out through face-to-face interviews by specifically trained interviews in the households of the respondents. As inclusion criteria, participants had to be older than 50, so a total sample of 461 participants was included in this study.

The ENSPG was originally designed to be comparable to the ENSE. For this reason, a stratified sampling by age and sex was used to ensure comparability between surveys. The questions and answers used in both questionnaires are the same, allowing their direct comparability between both populations, as well as their comparability over time, since these questions and answers have been widely used in population health surveys in Spain. The ENSPG questionnaire was designed based on that of the ENSE, maintaining the same order and wording of the questions.

Missing values did not exceed 1% of the data in both surveys, therefore were excluded from the analysis.

### Variables

#### Outcome variable: Functioning measure

The response variable considered was the GALI (Disabled/Not Disabled), collected through the same question in both health surveys: “For at least the past 6 months, to what extent have you been limited because of a health problem in activities people usually do?” It has three possible answers: Severely limited, limited but not severely, and not limited at all. The first two answers were grouped into a category representing those who were disabled, and the third answer represented those who were not disabled.

#### Context variables: Demographic and socioeconomic variables

To contextualize, demographic variables were used: sex (Male / Female) and age (50–64 / >65 years old). The following socioeconomic variables were also considered: employment status (Working / Unemployed / Retired / Others), educational level (No studies / Primary / Secondary / University) and household income (Low / Medium / High). Due to data source, household income was grouped differently in the Roma population (<950€ / 950–1950€ / >1950€ per month) and in the native and immigrant populations (<1050€ / 1050–1800€ / >1800€ per month).

#### Independent variables: Health conditions

The health conditions considered were grouped in the following categories for native and immigrant populations, according to the body function classification of the ICF: [1] cardiovascular diseases (myocardial infarction, coronary disease, other heart disease), arterial hypertension, neurological diseases (stroke, cerebral infarction, cerebral haemorrhage), musculoskeletal diseases (arthrosis, osteoporosis), respiratory diseases (asthma, chronic bronchitis, emphysema, chronic obstructive pulmonary disease), allergies, mental diseases (depression, anxiety, other mental problems), metabolic and endocrine diseases (high cholesterol, diabetes, thyroid problems), genitourinary diseases (urinary incontinence or urine control problems, kidney problems) cancer, sensory diseases (seeing problems, hearing problems), digestive diseases (stomach or duodenal ulcer, chronic constipation, cirrhosis, liver dysfunction, haemorrhoids), skin diseases (chronic skin diseases), chronic pain (chronic headaches, lumbar and cervical back pain) and accidents (being injured due to an accident at home, during leisure time or a traffic accident in the last 12 months). For the Roma population the health conditions were grouped considering the same classification: arterial hypertension, musculoskeletal diseases (arthrosis, arthritis and rheumatism and osteoporosis), respiratory diseases (asthma, chronic bronchitis, emphysema, chronic obstructive pulmonary disease), allergies, mental diseases (depression, other mental problems), metabolic and endocrine diseases (high cholesterol, diabetes), sensory diseases (seeing problems, hearing problems), digestive diseases (stomach or duodenal ulcer), chronic pain (chronic headaches) and accidents (having an accident of any kind in the last 12 months).

### Data analysis

To describe health conditions in all three populations, the prevalence of health conditions was calculated by population groups. An additional analysis by sex was calculated to include a sex perspective. To assess the contribution of health conditions to the prevalence of disability, the attribution method was used, calculating binomial additive hazard models [25,26]:

$$Y_{i}∼Bernoulli(\pi_{i})$$

$$\pi_{i}=1-e^{{-\eta }_{i}}$$

$$\eta_{i}=\alpha +\sum_{d-1}^{m}\beta_{d}X_{di}$$

This is a model where *Y*_*i*_ is the binary response variable for disability (disabled /not disabled) and *π*_*i*_ is the probability of being disabled for each individual i; *η*_*i*_ is the linear predictor (cumulative rate of disability) for each individual i; *α* is the background disability rate; *β*_*d*_ is the disability rate (disabling impacts) of each condition d; and *X*_*di*_ are the indicator variables for each chronic condition d and individual i. The main assumptions of this method are causality between health conditions and disability; all health conditions at the time of the survey and the background explain entirely the distribution of disability by cause; proportionality of this distribution with the risk of becoming disabled before the survey; simultaneity in the start of disability by cause; disability rates are similar for individuals over 50; causes of disability act as independent competing causes. Disabling impact rates, expressed as percentages, have been calculated with their 95% confidence intervals and p-values. Relative and absolute contribution of these rates to the prevalence of disability, expressed as a percentage, were also calculated. were fit using R (version 4.0.4) with the addhaz package.

### Ethics approval

The data were obtained from an anonymized database, based on administrative data obtained retrospectively, so no approval by an ethics committee is required.

## Results

### Contextualization and disability prevalence

This study has compared three different population groups in Spain: native, immigrant and Roma populations aged 50 and above. Age averages were different, being 60.3 years for immigrants, 65.9 years in the native population and 61.8 years in the Roma population. While native and Roma population are well balanced by sex, immigrant population is predominantly composed of females (59.2%). It is also remarkable that Roma people have the worst status in employment, household income and especially in education while immigrant population has a similar educational status and working status (mainly due to a low rate of retirement) than natives. However, immigrants have lower household incomes than natives (Table 1). As shown in Table 1, the prevalence of disability was higher in the Roma population (58.7%) and lower in the immigrant population (30.6%) than in the native population (39.4%).

**Table 1:** Demographic and socioeconomic variables and disability prevalence in native, immigrant and Roma populations.

Variables		Natives		Immigrants		Roma	
		n	%	n	%	n	%
**Sex **	Male	4670	47.3	322	40.8	225	48.8
	Female	5209	52.7	468	59.2	236	51.2
**Age **	50–64	5012	50.7	585	74.1	306	66.4
	65+	4866	49.3	204	25.9	155	33.6
**Employment status**	Working	2950	29.2	343	43.4	114	25.9
	Unemployed	697	7.1	132	16.8	70	15.9
	Retired	4581	46.4	196	24.9	83	18.9
	Other situations	1651	16.7	116	14.9	173	39.3
**Educational level **	No studies	288	2.9	25	3.2	124	27.3
	Primary	4341	43.9	183	23.2	322	70.9
	Secondary	5250	39.6	399	50.6	7	1.6
	University	1335	13.5	132	23.0	1	0.2
**Household Income **	Low	2416	32.7	233	41.6	325	83.8
	Medium	2455	33.2	164	29.3	57	14.7
	High	2525	34.1	163	29.1	6	1.5
**Disability**	Yes	3895	39.4	241	30.6	264	58.7
	No	5984	60.6	549	69.4	197	41.3

### Prevalence of chronic conditions

The prevalence of chronic conditions in the different groups, classified by the different body functions, was calculated by population group (native, immigrant, and Roma), as well as by sex. As shown in Table 2, arterial hypertension and metabolic diseases were almost the most prevalent conditions in the three population groups. In native and Roma populations the prevalence was respectively at 42.6% and 49.7% for arterial hypertension and 47.2% and 47.9% for metabolic diseases. The prevalence was lower in the immigrant population (30.9% for arterial hypertension and 38.9% for metabolic diseases). Indeed, in this population group, arterial hypertension was less prevalent than chronic pain (33.7%) and as prevalent as sensory problems (30.8%). Sensory problems were also noticed in the 48.6% of the Roma population and in the 41.3% of native population. In addition, musculoskeletal diseases were one of the most prevalent conditions in all three groups, but a significant lower prevalence was observed in the immigrant population (24.1%) compared to natives (38.9%) and Roma (41.4%). It can also be observed some wide disparities by sex in musculoskeletal diseases (24.4% in male vs. 49.7% in female), mental problems (12.7% in male vs. 26.9% in female), and in chronic pain (31.9% in male vs. 50.3% in female).

**Table 2:** Chronic conditions prevalence in native, immigrants and Roma populations.

Chronic conditions	Total Population				Native		Immigrants		Roma	
	Male		Female							
	n	%	n	%	n	%	n	%	n	%
**Cardiovascular**	879	17.6	729	12.8	1530	15.5	78	9.9	**	**
**Arterial hypertension**	2122	42.5	2327	41.0	4205	42.6	244	30.9	229	49.7
**Neurological**	204	4.1	158	2.7	334	3.4	25	3.2	**	**
**Respiratory**	512	10.2	805	14.2	1245	12.6	72	9.1	71	15.4
**Musculoskeletal**	1216	24.4	2821	49.7	3846	38.9	190	24.1	191	41.4
**Mental**	633	12.7	1529	26.9	2058	20.8	104	13.2	61	13.2
**Metabolic**	2248	45.0	2723	48.0	4664	47.2	307	38.9	221	47.9
**Genitourinary**	694	13.9	948	16.7	1553	15.7	89	11.3	**	**
**Digestive**	921	18.5	1351	23.8	2155	21.8	117	14.8	31	6.7
**Allergies**	594	11.9	989	17.4	1472	14.9	112	14.1	27	5.9
**Cancer**	334	6.7	517	9.1	809	8.2	41	5.2	**	**
**Accidents**	301	6.0	638	11.2	893	9.0	47	5.9	81	17.6
**Sensory**	1987	39.8	2334	41.1	4078	41.3	243	30.8	224	48.6
**Chronic skin**	387	7.8	458	8.1	806	8.2	39	5.0	**	**
**Chronic pain**	1594	31.9	2857	50.3	4184	42.4	266	33.7	99	21.5

**Variables not included due to data sources.

Immigrant population shows lower prevalence in all the categories. Compared to the native population, immigrants have lower prevalence in cardiovascular diseases (9.9% vs 15.5%), neurological diseases (3.2% vs 3.4%), respiratory diseases (9.1% vs 12.6%), genitourinary diseases (11.3% vs 15.7%), digestive diseases (14.8% vs 21.8%), cancer (5.2% vs 8.2%) and chronic skin problems (5.0% vs 8.2%). In the case of the Roma population, they had the highest prevalence of accidents (17.6%, compared to 9.0% in natives and 5.9% in immigrants) and respiratory diseases (15.4%). On the other hand, they had the lowest prevalence of allergies (5.9%, compared to 14.9% in natives and 14.1% in immigrants). Finally, mental health problems were more prevalent in the native population (20.8%) than in the immigrant (13.2%) and in the Roma (13.2%) populations.

### Disabling impact

As shown in Table 3, neurological and cardiovascular diseases had the highest disabling rates for both immigrants (93.94% [4.69–183.20] and 55.39% [25.41–85.37] respectively) and natives (59.10% [43.92–74.28] and 40.71% [34.89–46.54] respectively).

**Table 3:** Disabling impact of chronic conditions in native, immigrant and Roma populations.

Chronic conditions	Total Population						Native			Immigrants			Roma		
	Male			Female											
	Rate (%)	CI 95%	p-value	Rate (%)	CI 95%	p-value	Rate (%)	CI 95%	p-value	Rate (%)	CI 95%	p-value	Rate (%)	CI 95%	p-value
**Cardiovascular**	39.15	32.18–46.11	<0.001	45.69	35.81–55.58	<0.001	40.71	34.89–46.54	<0.001	55.39	25.41–85.37	<0,001	**		
**Arterial hypertension**	*			6.34	3.04–9.64	<0.001	3.22	1.19–5.294	<0.001	*			33.79	12.53–55.05	0.002
**Neurological**	68.38	47.89–88.87	<0.001	45.79	24.83–66.75	<0.001	59.10	43.92–74.28	<0.001	93.94	4.69–183.20	0.039	**		
**Respiratory**	23.81	15.39–32.24	<0.001	*			13.61	8.66–18.57	<0.001	*			*		
**Musculoskeletal**	31.00	25.10–36.91	<0.001	32.57	28.08–37.06	<0.001	33.56	29.86–37.26	<0.001	15.42	0.34–30.50	0.045	44.61	18.47–70.75	<0.001
**Mental**	43.40	34.17–52.63	<0.001	24.75	19.15–30.35	<0.001	32.11	27.14–37.07	<0.001	31.36	8.85–53.87	0.006	88.73	26.07–151.40	0.006
**Metabolic**	4.21	1.59–6.83	<0.001	4.34	1.41–7.27	0.007	3.93	1.88–5.97	<0.001	*			*		
**Genitourinary**	14.75	7.87–21.64	<0.001	40.73	31.77–49.70	<0.001	30.05	24.01–36.08	<0.001	*			**		
**Digestive**	9.52	4.32–14.72	<0.001	*			5.30	1.76–8.84	0.006	24.17	3.99–44.35	0.019	*		
**Allergies**	*			*			*			*			*		
**Cancer**	30.66	20.22–41.10	<0.001	29.42	20.31–38.52	<0.001	29.40	22.34–36.45	<0.001	45.85	9.87–81.84	0.013	**		
**Accidents**	8.91	1.07–16.74	0.026	23.75	15.31–31.98	<0.001	18.35	12.13–24.58	<0.001	*			105.54	55.82–155.25	<0.001
**Sensory**	10.17	6.95–13.39	<0.001	19.60	15.53–23.68	<0.001	13.53	10.92–16.14	<0.001	17.75	6.14–29.35	0.003	51.86	29.92–73.80	<0.001
**Chronic skin**	*			*			*			*			**		
**Chronic pain**	22.82	18.11–27.53	<0.001	22.27	18.11–26.44	<0.001	22.47	19.24–25.70	<0.001	23.05	9.62–36.48	<0.001	*		
**Background**	4.83	3.50–6.15	<0.001	3.82	2.47–5.16	<0.001	3.77	2.78–4.77	<0.001	8.30	4.70–11.89	<0.001	17.93	9.01–26.84	<0.001

*Statistically non-significant (p>0.05); The final model only included variables with significant adjusted effects.

**Variables not included due to data sources.

Immigrants and natives with a neurological disease had significant higher probabilities of having a disability than those with not neurological disease (p-values 0.039 and <0.001 respectively). In the case of cardiovascular diseases, the probabilities were higher for immigrants than for natives (55.39% [25.41–85.37] vs. 40.71% [34.89–46.54]). Musculoskeletal diseases also had a great disabling rate in the native population (33.56% [29.86–37.26]), but not in the immigrant population (15.42% [0.34–30.50]). The three health problems groups have a similar disabling effect in both male and female: Cardiovascular were at 39.15% [32.18–46.11] in males and 43.69% [35.81–55.58] in females; Neurological were at 68.38% [47.89–88.87] in males and 45.79% [24.83–66.75] in females; and Musculoskeletal were at 31.00% [25.10–36.91] in males and 32.57% [28.08–37.06] in females. On the other hand, the probability of being disabled due to mental health problems were similar for both natives (32.11% [27.14–37.06]) and immigrants (31.36% [8.85–53.87]), but significantly higher among male (43.40% [34.17–52.36]) compared to female (24.75% [19.15–30.35]), while with cancer the disability probability was higher among immigrants (45.85% [9.87–81.84]). In addition, digestive diseases also had a higher disabling rate for immigrants (24.17% [3.99–44.35]) than for natives (5.30% (1.76–8.84]) (not significant among women). Sensory problems and chronic pain also had significant disabling rates for both groups, being slightly higher for immigrants (17.75% [6.14–29.35] and 23.05% [9.62–36.48] respectively). In the case of sensory and genitourinary diseases, they had significantly higher disabling rates in female (19.60% [15.53–23.68] and 40.73% [31.77–49.79] respectively) than in male (10.17% [6.95–13.39] and 14.75% [7.87–21.64] respectively).

While only allergies and chronic skin problems were non-significant in the model for the native population (p>0.05), arterial hypertension, respiratory, metabolic, and genitourinary diseases, and accidents were non-significant in the immigrant population. Arterial hypertension and metabolic diseases had low disabling rates when adjusted with other conditions in the native population, but other conditions like genitourinary and respiratory diseases or accidents had a greater disabling rate in the native group.

Some chronic conditions were not included in the Roma model, so there are differences in specific conditions. While some conditions were found non-significant in the model (respiratory, metabolic, and digestive disease, allergies, and chronic pain), the cause-specific probability of disability were higher for the rest of the chronic conditions than in the other groups. In contrast to the other groups, accidents and mental health problems had the highest disabling rates (105.54% [55.82–155.25] and 88.73% [26.07–151.40]). Moreover, other conditions like sensory problems (51.86% [29.92–73.80]) and arterial hypertension (33.79% [12.53–55.05]) had higher disabling rates on this population group than on natives and immigrants. However, musculoskeletal diseases (44.61% [18.47–70.75]) had similar effects than in the native population.

### Contribution of chronic conditions

The models were used to obtain the relative and the absolute contribution of specific chronic conditions to the burden of disability in the three population groups. While relative contribution shows the effect in the prevalence of disability of each specific condition in the disabled population, absolute contribution shows the effect in the total population. So, as shown in Table 4, the sum of the effect of all specific conditions results in the disability prevalence of each group. The relative contribution was used in the primarily analysis because is easier to compare in groups with different disability prevalence.

**Table 4:** Contribution of chronic conditions to disability prevalence in native, immigrant and Roma populations.

Chronic conditions	Total Population				Native		Immigrants		Roma	
	Male		Female							
	Relative contribution (%)	Absolute contribution (%)	Relative contribution (%)	Absolute contribution (%)	Relative contribution (%)	Absolute contribution (%)	Relative contribution (%)	Absolute contribution (%)	Relative contribution (%)	Absolute contribution (%)
**Cardiovascular**	13.29	4.47	7.30	3.21	9.48	3.79	10.92	3.36	**	**
**Arterial hypertension**	*	*	3.99	1.75	2.48	0.99	*	*	16.42	9.50
**Neurological**	4.46	1.50	1.53	0.67	2.67	1.07	4.86	1.50	**	**
**Respiratory**	4.72	1.59	*	*	2.82	1.13	*	*	*	*
**Musculoskeletal**	14.78	4.97	23.44	10.30	20.89	8.35	8.62	2.65	16.77	9.71
**Mental**	10.02	3.37	9.35	4.11	10.24	4.10	8.74	2.69	8.63	5.00
**Metabolic**	4.31	1.45	3.27	1.44	3.33	1.33	*	*	*	*
**Genitourinary**	4.03	1.36	8.58	3.77	6.99	2.79	*	*	**	**
**Digestive**	3.67	1.23	*	*	1.92	0.77	7.94	2.44	*	*
**Allergies**	*	*	*	*	*	*	*	*	*	*
**Cancer**	3.98	1.34	3.77	1.65	3.78	1.15	5.08	1.56	**	**
**Accidents**	1.14	0.38	3.71	1.63	2.66	1.06	*	*	14.30	8.28
**Sensory**	8.93	3.01	11.89	5.22	9.63	3.85	13.25	4.08	23.84	13.80
**Chronic skin**	*	*	*	*	*	*	*	*	**	**
**Chronic pain**	15.04	5.06	16.69	7.33	15.82	6.33	18.29	5.63	*	*
**Background**	11.62	3.91	6.48	2.85	7.27	2.91	22.32	6.87	20.04	11.61
**Total**	100.00	33.65	100.00	43.92	100	40.00	100.00	30.79	100.00	57.90

*Statistically non-significant (p>0.05); The final model only included variables with significant adjusted effects.

**Variables not included due to data sources.

In the native population, musculoskeletal diseases were the higher contributors, representing the 20.89% of the disability burden. However, this problem mainly affects to female (it represents a half of the disability disparities by sex). Chronic pain and mental health problems were the following main conditions, contributing respectively 15.82% and 10.24% to the disability burden. Cardiovascular and sensory problems were also two important contributors, contributing 9.48% and 9.63% to disability prevalence. Genitourinary diseases contributed to 6.99% of disability and the rest of health conditions studied did it less than 5%.

In the immigrant population, chronic pain was the main contributor, being the cause of 18.29% of the disability prevalence in this group. In this group, sensory diseases contributed 13.25% and cardiovascular diseases 10.29% to the disability burden. On the other hand, musculoskeletal and mental diseases contributed respectively 8.62% and 8.74% to disability prevalence. Neurological diseases contributed 2 times more to the disability prevalence in this population group than in the native population (4.86% vs. 2.67%), and 4 times in the case of the digestive diseases (7.94% vs. 1.92%). Cancer contributed 5.08% to the disability burden in this group. In contrast to the native population, arterial hypertension, metabolic, respiratory, and genitourinary diseases, and accidents were no significant in the immigrant population.

In the Roma population, sensory diseases were the main contributors to the disability burden (23.84%). In contrast with the other groups, arterial hypertension and accidents contributed 16.42% and 14.30% to the disability prevalence. However, as for the native population, musculoskeletal diseases were an important contributor to disability burden (16.77%). On the other hand, mental health diseases contributed with 8.63%. As in the immigrant population, respiratory and metabolic diseases were no significant, however, chronic pain and digestive diseases were no significant in the Roma population.

## Discussion

Some health conditions have a higher disabling impact, regardless social factors. Health conditions like strokes or myocardial infarctions had already shown a great effect on disability in different populations [7,9]. It is important to understand that migrant and ethnicity status are not direct causes of disability. Physiological effects of certain pathologies may have similar effects in body functions among individuals, but context could determine whether a pathology leads to an impairment, and whether an impairment becomes a disability. In this sense, migrant and ethnicity could show patterns that can be employed to improve their health.

This study shows two general facts about the immigrant’s health: they have less prevalence of all health conditions, but chronic diseases are more disabling for them, especially the most severe ones. That means that although immigrant population have a better health status compared to the native population, which lead to a lower disability rate, among unhealthy people immigrants are more likely to be disabled. The first fact has been already reported in other countries [14] and different theories have tried to explain it [27]. On the other hand, the higher disabling impact of the health conditions has not been explained in previous studies. Structural xenophobia, including discrimination in the health care system, and the loss of social support when migrating may explain some of these disadvantages when developing a disease.

As found in previous studies, musculoskeletal diseases were the main contributor to disability burden, due to his high prevalence and disabling impact [7,28]. However, among immigrants the contribution was lower, mainly due to a lower disabling impact. This result has not been reported before, so new evidence will be needed to further explore it. Disparities by sex in this group of health problems could be an important factor. On the other hand, a higher severity and prevalence of musculoskeletal disease has been observed by ethnicity and race [29], although it has not been studied in Roma population. Some generalized habits in the Roma population, such as the use of inadequate footwear, could explain a higher prevalence of pain and musculoskeletal diseases. These cultural differences are related to a higher prevalence of foot health problems [30,31]. It must be noted that the model was not adjusted by back pain conditions in the Roma model, so musculoskeletal problems could include chronic pain conditions in participation restriction. In native and immigrant populations models, musculoskeletal diseases could explain restrictions in mobility while chronic pain could explain other restrictions related with those diseases [32].

In the case of cardiovascular, neurological diseases and cancer, their contribution to disability is mainly due to their great disabling impact. These results have been shown in previous studies for the general population in other European countries [7,9]. Furthermore, immigrant populations in other countries have shown a lower prevalence of these conditions [14,33]. Nevertheless, this study has found that these conditions may be more disabling among immigrants. Under-diagnosis and lack of knowledge could be at the origin of complications in those conditions [34]. In the case of the Roma population, these diseases have not been tested, but the high contribution of arterial hypertension in the burden of disability suggests that cardiovascular and neurological diseases are important contributors.

Mental health problems are one of the main contributors to the burden of disability in the three groups. Although the contribution was higher in the native population, mainly due to a higher prevalence, it could be an important disability cause in immigrant and Roma populations. In any case, it should be tested if there exists an under-estimation of mental health problems in this groups due to the social stigma associated with these conditions. In the USA migrant populations have shown an under-utilization of mental health services, with the particular importance of social support [35].

On the other hand, accidents had different results in native, immigrant and Roma populations. While it has shown to be the most prevalent and disabling condition among Roma, it has not been significant among immigrants and with a little contribution in the native population. However, immigrant population had shown a higher rate of occupational accidents [15], whereas ENSE did not include that kind of accidents. In the case of the Roma population, it was known that they have more traffic accidents and that this rate increased between 2006 and 2014, in spite of the implementation of the National Roma Integration Strategies (NRISs) 2020 [36]. More studies are needed to know the causes of this public health concern to apply more efficient focused measures.

This study has also provided evidence of other health conditions. Sensory diseases have contributed to disability burden in all three groups, especially in the Roma population. Among Roma, those limitations have been related with poor mental health and lower social participation [37]. Digestive diseases have also shown a great impact in the immigrant population. A study driven in the United States has found that immigration was associated with a loss of gut microbiome diversity, which could lead to digestive and metabolic diseases, such us obesity [38]. On the other hand, genitourinary diseases have not been significant in the immigrant model while it was an important contributor among natives. Finally, allergies and chronic skin diseases have not contributed to disability burden in any population group.

This study used a representative sample of the Spanish population, and it is the first study to assess the contribution of health conditions to disability by ethnic and migrant status; however, it has some limitations that must be noted. As a cross-sectional study, it is not possible to establish causality, despite the causality assumption of the attribution method. Furthermore, background contribution could be overestimated since important disability causes, such as dementia or occupational accidents, were not included, especially in the Roma population. Another limitation is due to data size, since it was not possible to stratify groups by age, sex, or educational level and by disability severity. In addition, there is some data source limitations, firstly because surveys have been collected in different years, which could slightly affect the comparability of the results. Secondly because most of the Roma population have born in Spain, so they could be included to some extent in the native population analyzed. Furthermore, two surveys with slight differences were used in this study, so some variables were not directly comparable.

## Conclusion

To conclude, migrant and ethnicity status are important factors in the disability burden. Life conditions and environment should be studied to develop preventive measures targeted at these specific populations. At the individual level, health care providers should establish strategies for the participation management of patients with cardiovascular and neurological diseases and cancer, because it could cause an increased participation restriction in social life. At a population level, public health measures should be taken to reduce burden of disability due to musculoskeletal diseases, chronic pain, and mental health conditions, in addition to cardiovascular diseases. Each group of diseases could be contributing to a particular participation restriction, so identify them could be useful to address effective measures. Barriers and facilitators should be addressed in the immigrant population to understand the factors that are causing these differences in disability. Other social variables, such as gender or country of birth, should be considered. The case of the Roma people should be considered separately, first because data collection is more complicate. This study has evidenced that sensory problems and accidents are at the origin of the 40% of the disability of this group. Public Health and institutions should consider the case of the Roma population and develop targeted strategies.
